# ICMR’s Antimicrobial Resistance Surveillance system (*i*-AMRSS): a promising tool for global antimicrobial resistance surveillance

**DOI:** 10.1093/jacamr/dlab023

**Published:** 2021-03-27

**Authors:** Jasmine Kaur, Ajay Singh Dhama, Harish Buttolia, Jasleen Kaur, Kamini Walia, Vinod Ohri, Vinit Kumar, Andrew M Lynn, Alok Srivastava, Harpreet Singh

**Affiliations:** 1 Division of Biomedical Informatics, Indian Council of Medical Research, New Delhi, India; 2 School of Computational and Integrative Sciences, Jawaharlal Nehru University, New Delhi, Delhi, India; 3 Amity Institute of Integrative Sciences and Health, Amity University Haryana, Amity Education Valley, Gurgaon, India; 4 Division of Epidemiology and Communicable Diseases, Indian Council of Medical Research, New Delhi, India; 5 Institute of Bioinformatics and Computational Biology, Visakhapatnam, Andhra Pradesh, India

## Abstract

**Background:**

Growing resistance to antimicrobials has become an important health issue of the 21st century. Many international, national and local approaches are being employed for the control and prevention of antimicrobial resistance (AMR). Among them, surveillance is reported to be the best method to reduce the spread of infection and thereby AMR. An integral component of AMR surveillance is the informatics suite for collection, storage and analysis of surveillance data.

**Methods:**

Considering the traits of an optimal surveillance tool and constraints with existing tools, Indian Council of Medical Research (ICMR) initiated the design and development of ICMR’s Antimicrobial Resistance Surveillance system (*i*-AMRSS). *i*-AMRSS is a web-based tool built using modular architecture. It is capable of collecting standardized data from small laboratories to generate local and nationwide reports.

**Results:**

*i*-AMRSS is a robust, comprehensive, modular, extensible and intelligent open-source tool piloted in ICMR’s AMR Network (31 hospitals and laboratories across India) since 2016. The developed tool has collected more than 280 000 patient records to date.

**Conclusions:**

The standardized data collected through *i*-AMRSS would be valuable for various collaborators to monitor outbreaks and infection control practices, evaluate transmission dynamics and formulate antibiotic use and selling policies. The tool is presently being used to capture human testing and consumption data, however, it can be extended for AMR surveillance using a ‘One Health’ approach.

## Introduction

Antimicrobial resistance (AMR) is a critical problem in the 21st century.^1^ Approximately 0.7 million people die every year worldwide from drug-resistant strains of microbes. The number is estimated to increase to 10 million by 2050, surpassing cancer (8.2 million deaths per year).^2^ In view of the increased resistance and drying pipeline of new antimicrobials, alternative approaches such as optimal use through surveillance, repurposing of antibiotics, regulation of antibiotic consumption and availability and improved hygiene and infection control have been endorsed.^1^^,^^2^ Among the available approaches, surveillance has been reported to be the best approach for reducing infection spread and thus AMR.^2^^,^^3^ It is believed that identification of resistance patterns and factors contributing to AMR, together with the reduced consumption of antimicrobials, may help in controlling the emergence and spread of AMR in pathogens.^4^

Realizing AMR’s severe threat, the World Health Assembly has formulated a global action plan (GAP) with five main objectives, one of which is to enhance knowledge and evidence through surveillance and research.^5^ Following the recommendations of the GAP, 63 member countries have, as per data available in November 2018, published national action plans (NAPs) for the containment of AMR. A comprehensive analysis of 52 NAPs (available in English) revealed that 44 NAPs considered establishing a national surveillance system. WHO’s global report on AMR surveillance^5^ also revealed gaps in AMR information in pathogens of major public health importance. Therefore, a comprehensive AMR surveillance system for collecting, storing and analysing the data on antibiotic resistance surveillance is essential.

Based on their scope, the existing surveillance tools can be classified into two groups: collectors and integrators. The collectors, like WHONET,^6^ are useful for individual laboratories to monitor AMR, whilst the integrators, like JANIS,^7^ collect data from multiple laboratory surveillance systems to generate nationwide reports. However, some limitations of the existing collectors and integrators include: (i) closed source with a limited capability for adding new modules; (ii) platform dependency with limitations on installation and entry of data from other platforms; (iii) local configurations with a limited ability to enforce uniform standards for data collection and analysis across the network; (iv) issues with data security, integrity, confidentiality and corruption in shared computational resource settings; (v) limited client-server architecture; and (vi) lack of data validation.

**Figure 1.**
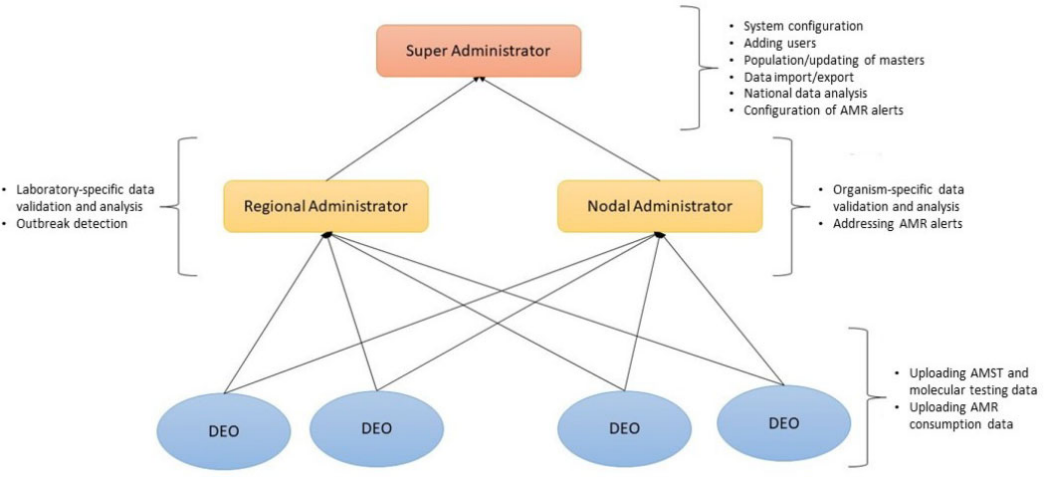
The data flow and four major functional modules of *i*-AMRSS (DEOs, regional administrator, nodal administrator and super administrator).

Thus, a hybrid solution with a comprehensive system bridging laboratories and hospitals is urgently required to surmount these constraints in order to project a complete picture of AMR in the country.^8^ In this paper we present an overview of Indian Council of Medical Research (ICMR)’s Antimicrobial Resistance Surveillance system (*i*-AMRSS), a promising tool for collection, management and analysis of the AMR data. The tool has been developed using open-source technologies and can be implemented at all levels: laboratory and institutional as well as national. A flow diagram depicting the working modules for *i-*AMRSS data collection is shown in Figure 1.

## Methods

### System architecture

*i*-AMRSS is a Linux-based software package/tool for management and analysis of antimicrobial susceptibility testing and antimicrobial consumption data collected through multiple laboratories across India. The system is hosted on the Ubuntu 16.04 operating system using Apache web server. The design and development of *i*-AMRSS includes the use of latest web technologies such as PHP 7, JavaScript, AJAX, etc. as frontend tools and a MySQL (Version 5.6) database as a backend software tool. The tool has been developed in a modular architecture with the prevailing version offering various features to the users: (i) uniform data collection and validation modules (including an administrative module for configuring users, hospitals, laboratories, antimicrobials, organisms, antimicrobial susceptibility test panels, etc.); (ii) offline data entry through Excel; (iii) detailed analysis at both the laboratory and national level; (iv) application programming interface (API)-based data sharing (both import and export)—the data structure is compatible with major databases and statistical software programs; (v) dashboards for different stakeholders; (vi) security and audit trail; (vii) molecular data collection and analysis; (viii) social media interface for public awareness; and (ix) data integration and big-data analytics.

### Data source

The *i*-AMRSS tool is currently used to collect microbiology data from 31 tertiary care hospitals and laboratories across India. The system has provision to capture the data generated by different test methods, such as disc diffusion, MIC assay and automated testing methods. In the system, the numeric values (for disc diffusion/MIC assay) are captured which are interpreted using the pre-defined breakpoints (CLSI/EUCAST guidelines). This tool was launched in 2016 in the initial phase to four tertiary care hospitals; it was later extended and now has more than 280000 patient records.

The demographic details of the patient, hospital information and sample information are also collected along with the resistance data. Currently, the antimicrobial susceptibility testing (AMST) data for 55 antibiotics and antifungals, tested against 116 commonly isolated bacterial and fungal species, isolated from 80 different types of human samples are collected and analysed. The system is fully customizable and scalable to include any number of antimicrobials, organisms and samples. The system also has a provision to collect the total samples received (denominator data) for analytical purposes.

### Data analytics

**Figure 2.**
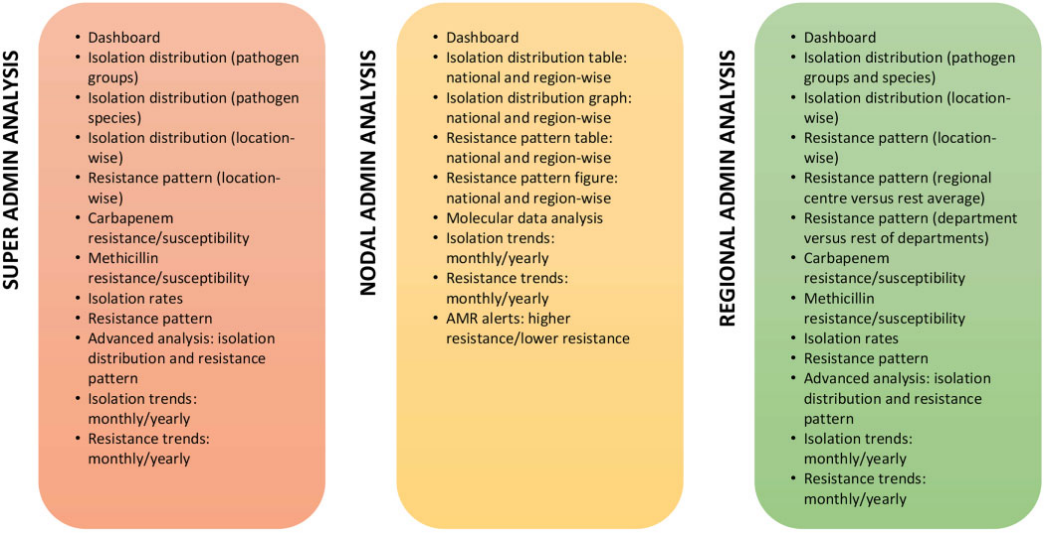
The analysis options available in the current version of the tool. A detailed description of each option is available in Table S1.

The data analytics module enables users to extensively analyse the data stored in the system. The analysis is provided to the user in the form of pie charts, bar graphs, stack bar graphs and tables depicting comparative and individual isolation rates and resistance patterns across samples, species and locations. The tables were developed using HTML and graphics were built using D3 library charts.^9^Figure 2 outlines the analysis options available in the current version of *i*-AMRSS.

### Data sharing

The accessibility permissions and data sharing at different levels/modules is shown in Figure S1 (available as Supplementary data at *JAC-AMR* Online). The organism-specific data are shared with the nodal administrator who can view the inter-region data pertaining to a specific organism collected from multiple centres/labs. The laboratory-specific data collected from multiple centres/labs within that regional centre are shared with the regional administrator of that site. The entire database is shared with the super administrator. The data in the system are not yet publicly available. However, the reports generated from the data are annually updated on the ICMR’s Antimicrobial Resistance website (iamrsn.icmr.org.in).^10^

### Ethics

This study includes the 31 hospitals that are part of the AMR surveillance and research initiative of ICMR. These sites routinely share AMR data for clinical isolates received by clinical microbiology labs and capture antimicrobial prescriptions and clinical outcomes of patients with drug-resistant infections. The study does not require taking informed consent from all patients for which AMR data are added to the ICMR data management system as (i) no patient is sampled exclusively for project work; (ii) all samples are collected as part of standard of care for patient management; and (iii) the data are utilized maintaining full confidentiality after removing all the patient identifiers.

## Results

*i*-AMRSS is a fully customizable tool for collection, management, analysis and reporting of the AMR data. It was developed by ICMR as part of ICMR-AMR Surveillance Network to enforce quality AMST in laboratories.

*i*-AMRSS has been proven to be really valuable by providing comprehensive analytics to support research and development of guidelines on the use and control of antimicrobials for public consumption. Additionally, annual reports are published online, and anonymized data are also reported to the Global Antimicrobial Resistance Surveillance System through the designated National Coordinating Centre for India at National Centre for Disease Control (NCDC), New Delhi. Being a web-based tool, it has minimal hardware requirements and can be easily integrated into the existing laboratories in the country. A standalone version of the tool will be provided for laboratories where there are restricted computational resources and/or no internet facilities. Some of the salient features of the tool are as follows.

### Role-based access

*i*-AMRSS allows each user to access the required information based on their role as shown in Figure S1. There are five categories of user [super administrator, nodal administrator, regional administrator, data entry operator (DEO) and user or policy-maker]. Customized modules have been designed for each user to ensure data security and confidentiality.

### Enforcing quality AMST through standardization in data collection (super administrator module)

The super administrator module allows the super administrators to configure the system. System configuration includes master tables (Figure S2) to add or update antibiotics, hospitals, laboratories, organisms, samples and organism-specific antimicrobial panels (a set of antimicrobials and molecular tests for a given organism or organism group that has been cultured from a given clinical sample). This enforces uniformity and standardization in AMST across the network. Furthermore, the super administrator can create and update different users under the network by assigning them laboratories/organisms as per their role. For example, a super administrator specifies the set of organisms to be validated by the nodal administrators and the laboratories that come under the regional centre. Additionally, the super administrator can configure AMST alerts for unusual antibiotic test results obtained for any organism. Records with such alerts are highlighted when they appear to the nodal administrator for validation, allowing the nodal administrator to take action as necessary.

### Data collection module

The data collection module is flexible, as data can be entered through the online platform using a built-in integrated form (Figure S3) or uploaded using Excel. The online entry form has four parts: (i) patient information; (ii) hospital information; (iii) sample information; and (iv) susceptibility test values. All the system attributes configured by the super administrator are visible in this form. The form can be reset at any of the above parts, allowing the entry of multiple samples and multiple organisms with ease. The data entry process is compact and user-friendly (Figure S4). The status of all the records entered by the DEO can be seen in the menu bar under ‘records to be validated’, ‘accepted records’, ‘rejected records’ and ‘records for revision’ (Figure S3). The DEO can edit the records pending for revision from the menu bar. The Microsoft Excel offline data entry module allows the DEO to upload the required data via a predefined Excel format, which is optimal primarily for areas with poor and intermittent internet connectivity.

### Data validation module

The nodal administrators are experts who validate all the data uploaded into *i*-AMRSS for each group of organisms. When the super administrator creates a new account, each expert is assigned to a group of organisms. All the data entered for those organisms are sent to the nodal administrator for validation. The nodal administrator dashboard provides regional centre-wise statistics for all records uploaded (Figure S5). The AMST results appear colour-coded as resistant, intermediate and susceptible based on the respective cut-off values, as depicted in Figure S6. The alerts for unusual resistance patterns in any organism are also visible. Thus, no unusual patterns are missed out, thereby providing insights into future outbreaks. During validation, the nodal administrator can accept, reject or send the record back to the DEO for revision. A module for automated validation of records (using machine learning techniques) will be released soon.

### Data analytics

**Figure 3.**
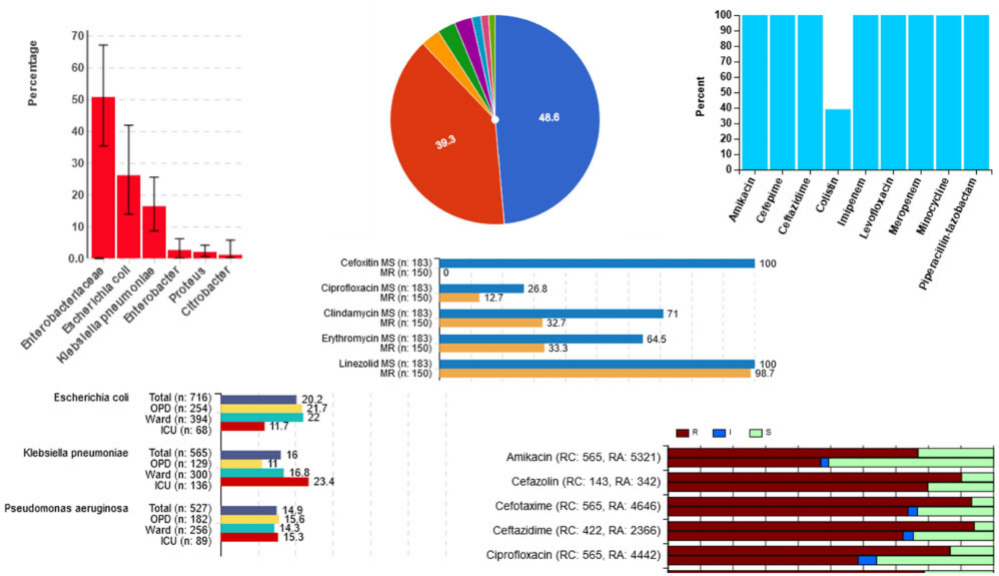
The different graphics available in the current version of *i*-AMRSS.

Data analytics is one of the most essential and useful features of *i*-AMRSS. There are different data analytics screens for each stakeholder or user of the system. The laboratory-based analyses are performed by the regional administrator. The dashboard for the regional administrator analysis module is shown in Figure S7. The menu in this dashboard comprises multiple options that enable the regional administrator to comprehensively analyse all the data entered into the system. The analysis can be performed on the data for a specific time period and for one or multiple laboratories under the regional administrator. The findings can then be interpreted for a laboratory using horizontal and vertical bars, pie charts and stacked bar graphs, as shown in Figure 3.

**Figure 4.**
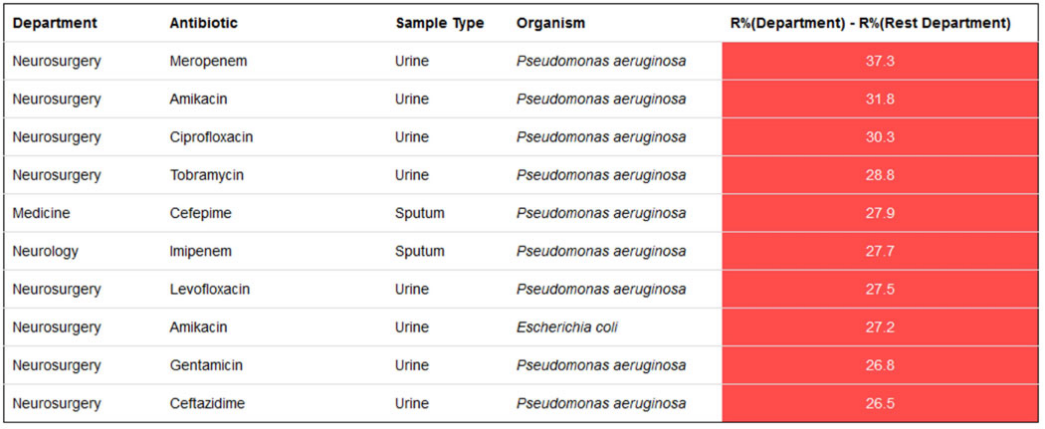
Alerts showing higher resistance in the specified departments of the regional centre compared with other departments.

Some of the available analytics options include: (i) graphs depicting the sample-wise isolation distribution of all the organism groups and species (Figure S8); (ii) sample-wise isolation of the top 10 organism species from different hospital locations (Figure S9); (iii) comparison of the percentage of organisms isolated from different locations for a selected sample type (Figure S10); (iv) location-wise percentage resistance to tested antibiotics for different organism species (Figure S11); (v) sample-wise comparison of the antibiotic resistance pattern in a regional centre versus the average across the rest of the centres (Figure S12); (vi) sample-wise comparison of the antibiotic resistance pattern in a hospital department versus the average across the rest of the departments (not shown, but similar to Figure S12); (vii) pattern of resistance to other antibiotics in carbapenem-resistant and -susceptible strains (Figure S13); (viii) pattern of resistance to other antibiotics in methicillin-resistant and -susceptible strains; and (ix) percentage of antibiotics tested in the user defined antibiotic panels (Figure S14). Tables for sample-wise and location-wise isolation rates (Figure S15, Figure S16) and resistance patterns (Figure S17) are also generated. Except for the fixed tables and graphs, there is an advanced analysis option in which the regional administrator can generate tables based on different permutations and data combinations entered in the portal (Figure S18). The system also gives alerts on higher and lower resistance, which can be used to monitor the effectiveness of the infection control practices in each department (Figure 4) and regional centre. Alerts for higher resistance are generated using a combination of samples, antibiotics and organisms in the selected department compared with the average in other departments in the regional centre. Resistance 10%–20% higher is colour-coded orange and resistance ≥20% higher is colour-coded red. Similar alerts are available for each regional centre compared with other centres in the network. Alerts for lower resistance information are also provided for each regional centre and department.

The nodal administrator completes an organism-specific analysis of the data entered from various laboratories. The dashboard for nodal centre analysis can be seen in Figure S19. The analysis can be performed for all data or a specific time period. Some of the available analytics screens are graphs and tables for isolation rates for user-selected sample types across different regional centres (Figure S20 and Figure S21), percentage resistance to antibiotics tested for different organism species across different regional centres (Figure S22 and Figure S23), resistance patterns in carbapenem-resistant and -susceptible strains, resistance patterns in methicillin-resistant and -susceptible strains, and percentages of positive phenotypic and genotypic tests across all the regional centres (Figure S24). The system also gives alerts on higher and lower resistance, which can be used to monitor the effectiveness of the infection control practices for the selected organism species in each regional centre (Figure S25).

Furthermore, there are dashboards that depict a nationwide picture by comprehensively analysing all the data in the network. These can be leveraged by the policy-makers to support development of national guidelines on antimicrobial usage and testing.

### Antibiotic consumption module

The antibiotic consumption module is an Excel form that collects antibiotic consumption information from various hospitals. We developed forms based on defined daily dose (DDD) and days of therapy (DOT) guidelines by WHO for collection of antimicrobial consumption data on a daily, weekly, monthly and yearly basis.^11^

### Data import and export

An API has been developed to import and export data in the network.

### Data security and confidentiality

*i*-AMRSS ensures data security through role-based access, where each stakeholder can only view the screens permitted for their respective role. Additionally, an audit trail is maintained for each record entered and validated in the developed tool. Sensitive information, such as address and mobile telephone number, is not captured in the *i*-AMRSS system. Data security is maintained with strong encryption of data (patient ID and sample ID) in the server.

## Discussion

India is one of the major contributors to AMR’s global burden. This can be attributed to the high prevalence of antimicrobial overuse, abuse and misuse throughout the country.^12^ Contrary to developing newer drugs, surveillance methods have been found to be effective in controlling antibiotic resistance.^13^ Some countries (like Japan) have developed their own national surveillance tools to restrain AMR, but no similar tool has been developed in India to date.^14^ We present an overview of the first software tool, named *i*-AMRSS, designed and developed in India with excellent efficiency for collecting and analysing AMR data across the country.

*i*-AMRSS is a collective package for electronic surveillance of AMR information entered by DEOs in multiple regions in India on a case-by-case basis. The distribution of authority and accessibility permissions at multiple levels—super administrator, regional administrator and nodal administrator—ensures stringent data validation, a feature that makes *i*-AMRSS unique when compared with other AMR surveillance tools worldwide. In addition to data collection, *i*-AMRSS includes an integrated reporting module that facilitates real-time analysis of the data entered in the portal. The analysis package includes modules for organism-specific, laboratory-specific and national-level data analysis. In addition, an advanced analysis feature enables the ability to compare and analyse all the data. Today, *i*-AMRSS offers India’s first web-based analytics platform for AMR surveillance. Moreover, the automated system used in *i*-AMRSS analysis monitors any irregular patterns in organisms isolated from various specimens at two levels providing warnings of high resistance and low resistance. Comprehensively, *i*-AMRSS provides reliability, timeliness and standardization for the collection, management and analysis of AMR data. The tool will set standards for laboratory, hospital and nationwide AMST practices.

*i*-AMRSS’s flexibility to expand the list of organisms for any given region reflects its scalability, which gives it an advantage over other existing tools. Some of the future directions in this study include integrating the modules for predictive analysis, identification of outbreaks and dissemination of data exchange standards among different surveillance tools. Additionally, the tool will be released as a standalone package to be used in small laboratories across India. Since the module for antibiotic consumption is still in the data collection stage, correlation with AMST data is yet to be developed. Furthermore, making the data publicly available with the right policies and standards is another improvement that will be integrated in version 2 of *i*-AMRSS.

## Supplementary Material

dlab023_Supplementary_Data
