# Supplementary material for: ICMR’s Antimicrobial Resistance Surveillance system (i-AMRSS): a promising tool for global antimicrobial resistance surveillance
Source: JAC Antimicrob Resist. 2021 Mar 27;3(1):dlab023. doi: 10.1093/jacamr/dlab023 (PMC8210178; doi:10.1093/jacamr/dlab023)
Supplement: dlab023_Supplementary_Data [file dlab023_supplementary_data.docx]

**Supplementary data**

**Table S1:** Table describing all the available analysis options in the Super admin, regional centre and Nodal centre modules.

| Type of Analysis | Description | Example |
| --- | --- | --- |
| Super Admin Analysis | **Analysis of all the data in the network.** |  |
| Dashboard | Bacteria group-wise number of uploaded & validated records. Provides option for the selection of laboratories and duration of analysis. | Similar to Figure S7 |
| Isolation Distribution (Pathogen groups) | Pie chart depicting sample-wise (include abundantly occurring sample types only) isolation distribution of different pathogen groups. Distribution can be seen up to species level. | Similar to Figure S8 |
| Isolation Distribution (Pathogen species) | Pie chart depicting user-selected sample and location-wise isolation percentages of top 10 isolates. | Similar to Figure S9 |
| Isolation Distribution (Location-wise) | Bar charts depicting location-wise distribution of top 10 isolates for the user-selected sample types. | Similar to Figure S10 |
| Resistance Pattern (Location-wise) | Bar chart depicting location-wise resistance pattern of selected isolate in different health care areas for user-selected sample types. | Similar to Figure S11 |
| Carbapenem resistant(CR) / Carbapenem susceptible(CS) | Bar charts depicting resistance pattern by Carbapenem resistance and susceptible strains against other antibiotics. | Similar to Figure S13 |
| Methicillin-resistant(MR) / Methicillin-susceptible(MS) | Bar charts depicting resistance by Methicillin resistance and susceptible strains against other antibiotics. | Similar to Figure S13 |
| Panel-wise percentage of Antibiotics Tested | Bar charts depicting percentage of antibiotics tested in user-selected antibiotic panel. | Similar to Figure S14 |
| Isolation Rates | Tables depicting sample-wise and location-wise isolation rates of different pathogen groups and species. | Similar to Figure S15 |
| Resistance Pattern | Tables depicting sample-wise and location-wise resistance pattern of different pathogen groups and species. | Similar to Supplementary Figure |
| Advance Analysis - Isolation rates and Resistance pattern | Options to generate tables for isolation rates and resistance percentages using different combinations of data available in the network. | Similar to Figure S18 |
| Regional Centre Analysis | **Analysis of all the data uploaded from multiple laboratories in the regional centre.** |  |
| Dashboard | Bacteria group-wise number of uploaded & validated records. Provides option for the selection of laboratories and duration of analysis. | Figure S7 |
| Isolation Distribution(Pathogen groups) | Pie chart depicting sample-wise (include abundantly occurring sample types only) isolation distribution of different pathogen groups. Distribution can be seen up to species level. | Figure S8 |
| Isolation Distribution(Pathogen species) | Pie chart depicting user-selected sample and location-wise isolation percentages of top 10 isolates. | Figure S9 |
| Isolation Distribution(Location-wise) | Bar charts depicting location-wise distribution of top 10 isolates for the user-selected sample types. | Figure S10 |
| Resistance Pattern(Location-wise) | Bar chart depicting location-wise resistance pattern of selected isolate in different health care areas for user-selected sample types. | Figure S11 |
| Resistance Pattern (Regional Centre vs Rest Average) | Stacked bar charts depicting comparative account of Resistance and Susceptibility in the regional centre as compared to rest average. | Figure S12 |
| Resistance Pattern (Department vs Rest Department) | Stacked bar charts depicting comparative account of Resistance and Susceptibility in a department of regional centre as compared to rest departments. | Similar to Figure S12 |
| Carbapenem resistant(CR)/Carbapenem susceptible(CS) | Bar charts depicting resistance pattern by Carbapenem resistance and susceptible strains against other antibiotics. | Figure S13 |
| Methicillin-resistant(MR)/ Methicillin-susceptible(MS) | Bar charts depicting resistance by Methicillin resistance and susceptible strains against other antibiotics. | Similar to Figure S13 |
| Panel-wise percentage of Antibiotics Tested | Bar charts depicting percentage of antibiotics tested in user-selected antibiotic panel. | Figure S14 |
| Isolation Rates | Tables depicting sample-wise and location-wise isolation rates of different pathogen groups and species, | Figure S15, Figure S16 |
| Resistance Pattern | Tables depicting sample-wise and location-wise resistance pattern of different pathogen groups and species. | Figure S17 |
| Advance Analysis - Isolation rates and Resistance pattern | Options to generate tables for isolation rates and resistance percentages using different combinations of data available in the network. | Figure S18 |
| Alerts - Regional Centre vs Rest Average- Higher Resistance Alerts | Checks in a combination of all samples, antibiotics and organism for higher resistance in the selected Regional centre as compared to Rest. 10-20% Higher resistance is color coded orange and greater than 20% resistance is color coded red. | Figure 4 |
| Alerts - Regional Centre vs Rest Average- Lower Resistance Information | Checks in a combination of all samples, antibiotics and organism for lower resistance in the selected Regional centre as compared to Rest. 10-20% Lower resistance is color coded light green and greater than 20% resistance is color coded dark green. | Similar to Figure 4 |
| Alerts - Department vs Rest Department- Higher Resistance Alerts | Checks in a combination of all samples, antibiotics and organism for higher resistance in a particular department of the selected Regional centre as compared to Rest departments in the regional centre. 10-20% Higher resistance is color coded orange and greater than 20% resistance is color coded red. | Similar to Figure 4 |
| Alerts - Department vs Rest Department- Lower Resistance Information | Checks in a combination of all samples, antibiotics and organism for lower resistance in a particular department of the selected Regional centre as compared to Rest departments in the regional centre. 10-20% Lower resistance is color coded light green and greater than 20% resistance is color coded dark green. | Similar to Figure 4 |
| Nodal Centre Analysis | **Analysis of all the data uploaded for an organism group.** |  |
| Dashboard | Bacteria group-wise number of uploaded & validated records. Provides option for the selection of laboratories and duration of analysis. | Figure 19 |
| Isolation Rates-Table | Table depicting isolation rates of different pathogen groups and species isolated from selected sample and location. | Figure 20 |
| Isolation Rates-Graph | Graph depicting isolation rates of different pathogen groups and species isolated from selected sample and location. | Figure 21 |
| Resistance Pattern-Table | Table depicting resistance pattern of different pathogen groups and species isolated from selected sample and location. | Figure 22 |
| Resistance Pattern-Graph | Graph resistance pattern of different pathogen groups and species isolated from selected sample and location. | Figure 23 |
| Carbapenem resistant(CR) / Carbapenem susceptible(CS) - Table | Tables depicting resistance percentage in other antibiotics in Carbapenem resistance and susceptible strains isolated in different regional centres. |  |
| Carbapenem resistant(CR) / Carbapenem susceptible(CS) - Graph | Bar charts depicting resistance in other antibiotics in Carbapenem resistance and susceptible strains isolated in different regional centres. |  |
| Phenotypic/Genotypic - Tables | Tables depicting for positive percentages of Phenotypic and genotypic tests for mechanism of resistance in different organism species | Figure S24 |
| Alerts - Regional Centre vs Rest Average- Higher Resistance Alerts | Checks in a combination of all samples, antibiotics and organism for higher resistance in all Regional centre as compared to Rest. 10-20% Higher resistance is color coded orange and 20% and above resistance is color coded red. | Figure S25 |
| Alerts - Regional Centre vs Rest Average- Lower Resistance Information | Checks in a combination of all samples, antibiotics and organism for lower resistance in all Regional centres as compared to Rest. 10-20% Lower resistance is color coded light green and 20% and above resistance is color coded dark green. | Similar to  Figure S25 |

**
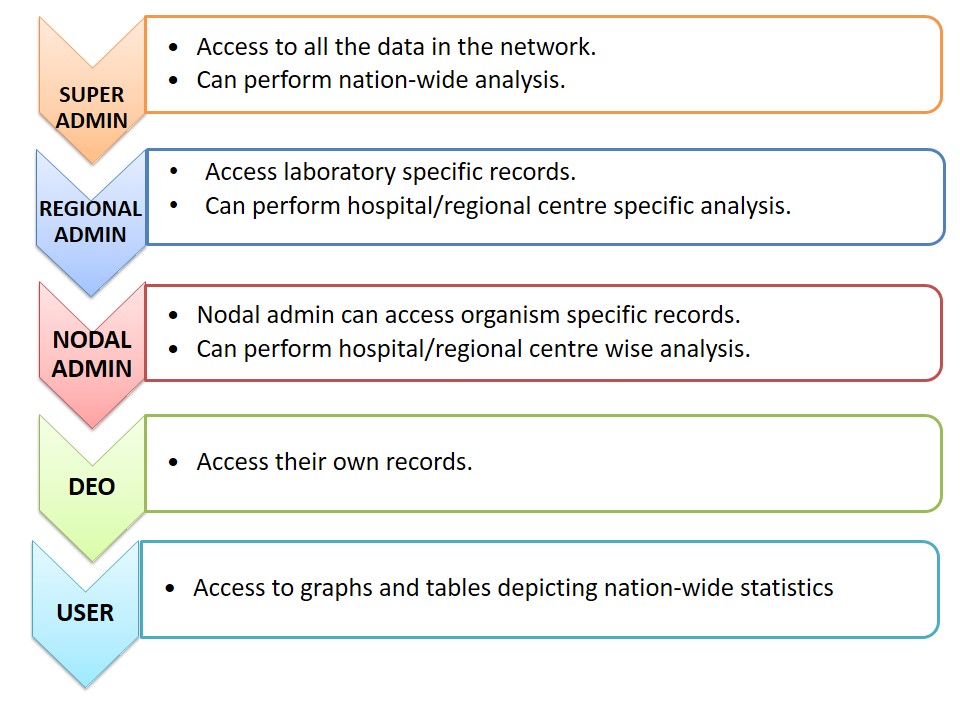
**

**Figure S1**: Figure depicting the roles of different categories of users in *i-*AMRSS and their access to data available in the network.


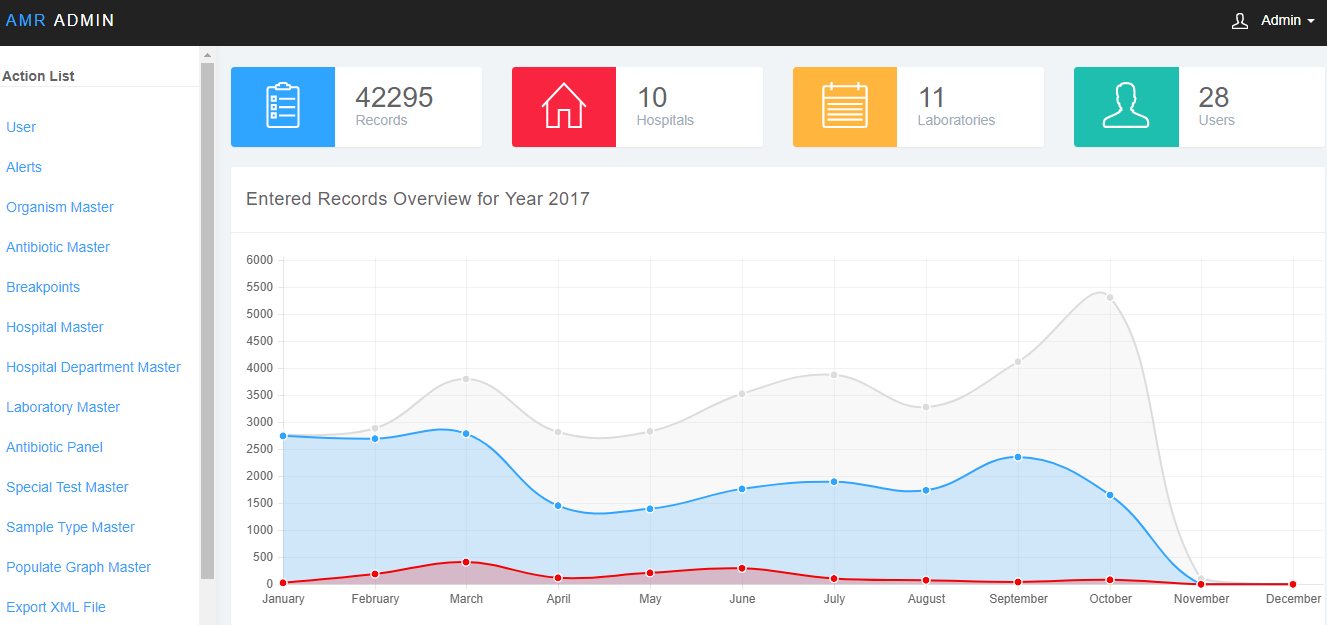


**Figure S2**: Figure depicting dashboard for the super-admin module. Four boxes on the top describe the total number of records, number of hospitals, laboratories and users in the network. Dashboard also consists of a graph depicting monthwise statistics of uploaded and validated records. All the master tables are available in the left panel under the Action list.


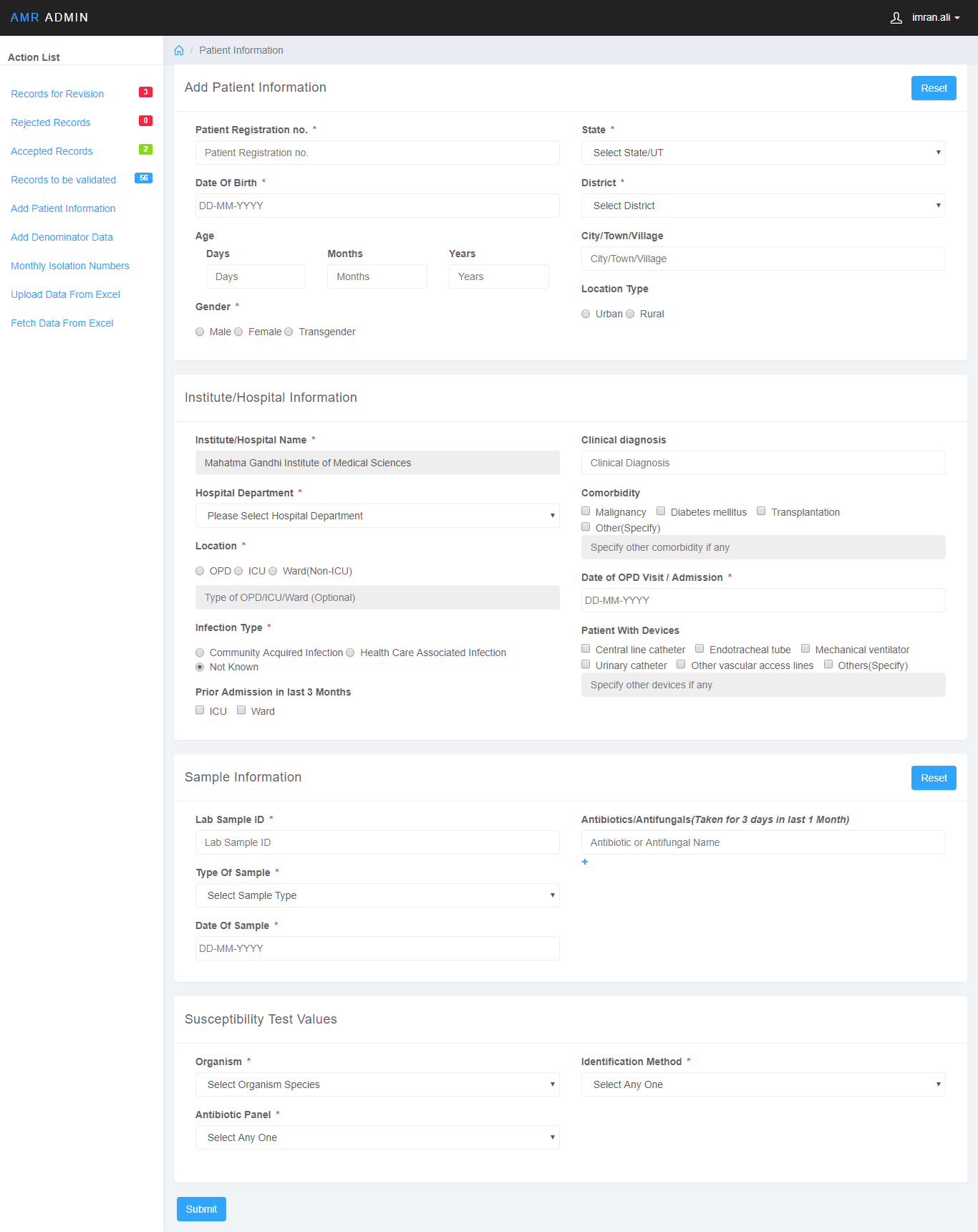


**Figure S3**: Figure depicting online data entry module. It comprises of a built-in form comprising four parts; patient information, hospital information, sample information and susceptibility test values. All the attributes of the system configured by the super administrator are visible in this form. After successful submission of the form, the record is visible under one of the four links (Accepted records, rejected records, records for revision and Records pending validation) available in the Action list. Two other available menu options include uploading denominator data and monthly isolation numbers.


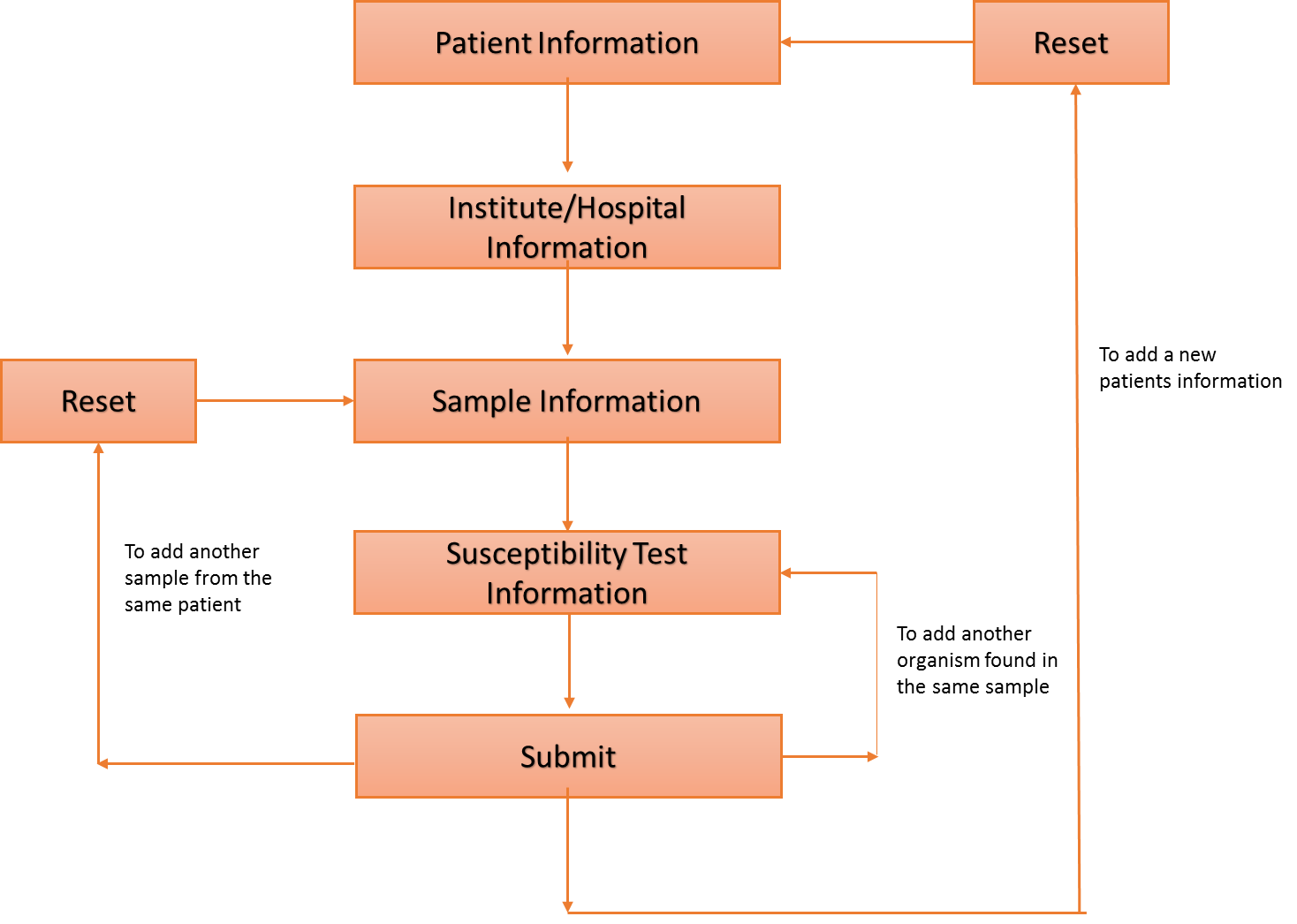


**Figure S4**: Flow diagram depicting the online data entry process. The data entry form comprises of four parts (Patient information, Hospital information, Sample information and Susceptibility test information). To add more than one sample of a patient, the form can be reset at Sample Information part.


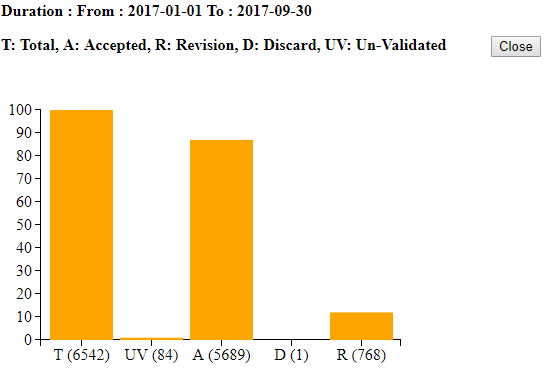


**Figure S5**: Figure depicting statistics for total uploaded, accepted, discarded and un-validated by Nodal centre. This is available for all the Nodal centres.


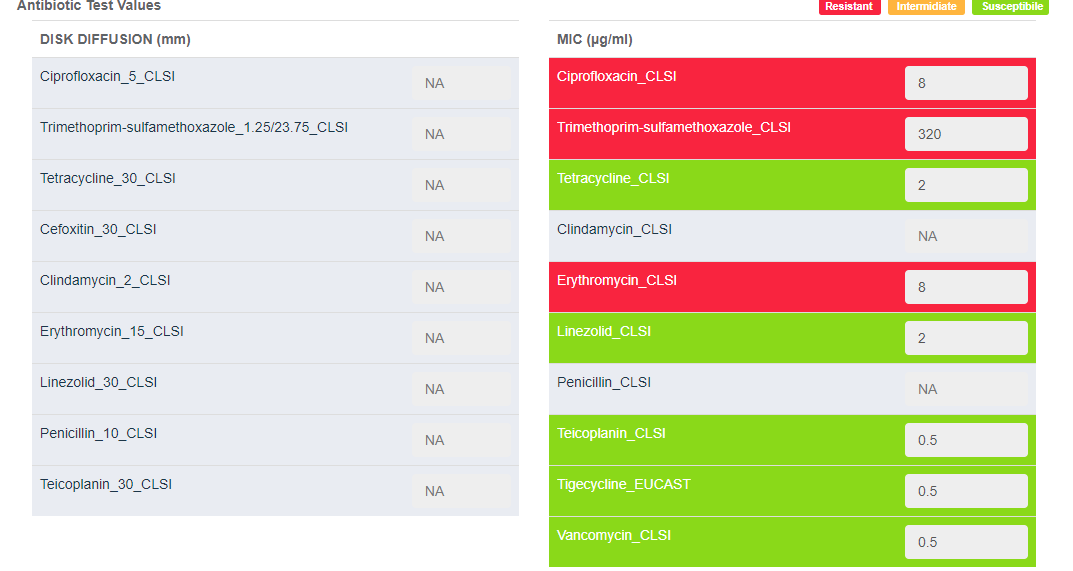


**Figure S6**: Figure depicting colour coded records. Resistant values are coloured red, intermediate orange and susceptible green.


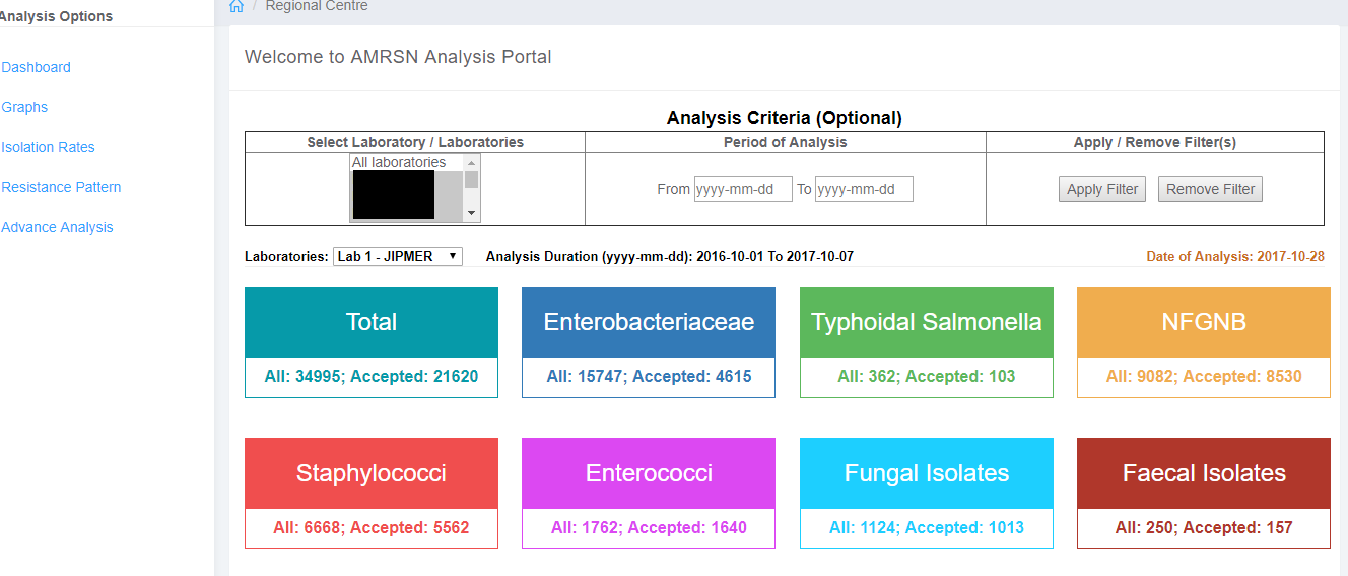


**Figure S7**: Figure depicting dashboard for regional centre analysis module. Broader categories of the available analysis options are shown in the left panel. Dashboard provides statistics for organism group-wise records uploaded and accepted from the regional centre. Options to select laboratories and duration for analysis is also available on the dashboard.


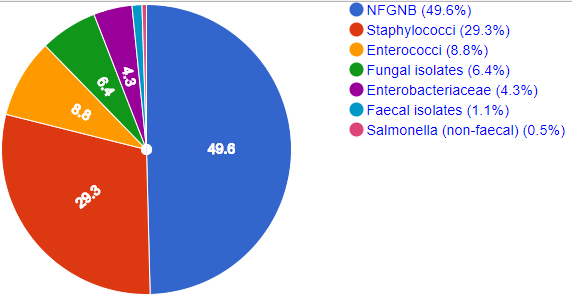


**Figure S8**: Pie chart depicting isolation distribution of records in different organism groups isolated from all positive cultures. Each partition is clickable and gives distribution of records in organism species within that group.


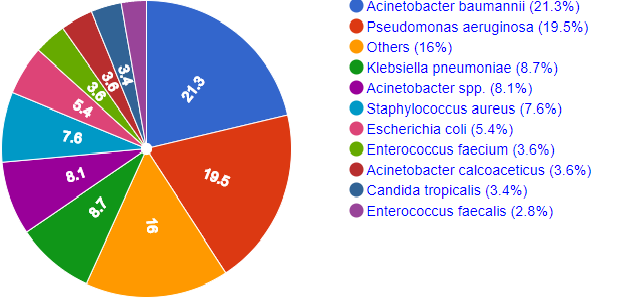


**Figure S9**: Pie chart depicting distribution of records in top 10 isolates in all samples taken from ICU. This graph can be generated for a combination of any sample and location type.


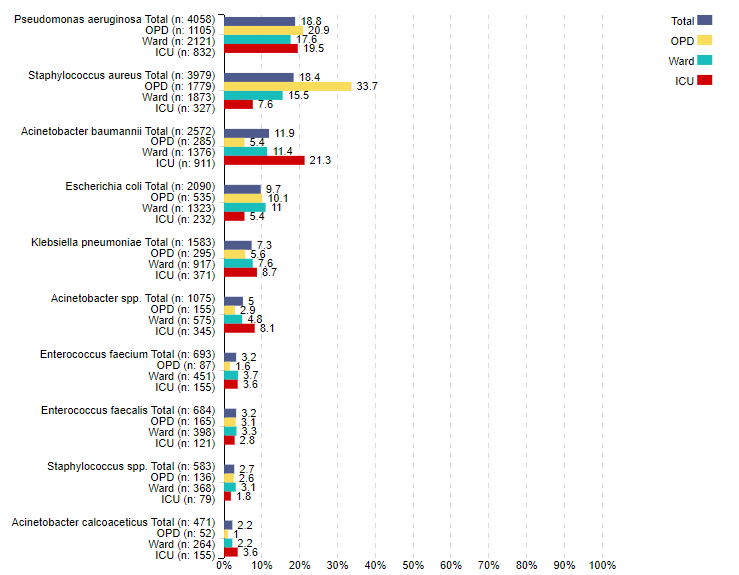


**Figure S10**: Bar charts depicting isolation percentages of top 10 isolates taken from all samples across different locations. In each set blue, yellow, green and red colored bars represent the isolation percentage of organism species from selected sample taken from all locations, OPD, ward and ICU. This graph can be generated for any sample type in the system.


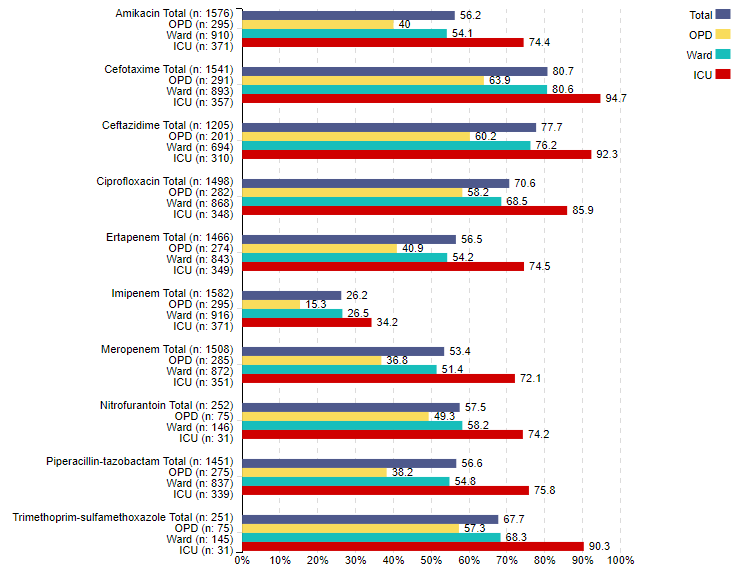


**Figure S11**: Bar charts location-wise resistance percentage of antibiotics tested for Klebsellia pnemonie isolated from all samples. In each set blue, yellow, green and red colored bars represent the isolation percentage of organism species from selected sample taken from all locations, OPD, ward and ICU. This graph can be generated for any sample type and any organism species.


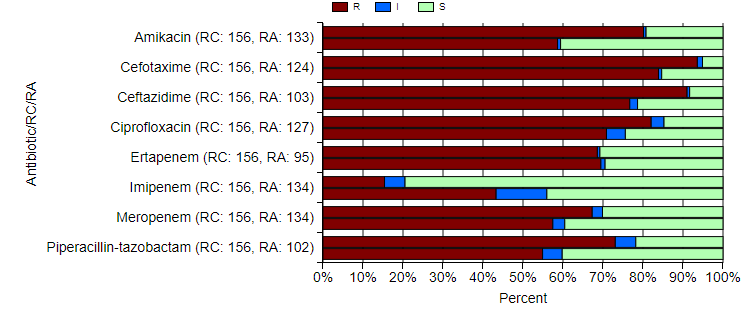


**Figure S12**: Stacked bar charts giving a comparative account of RIS in a regional centre as compared to rest regional centres in the network. For each antibiotic upper bar represents the regional centre and lower bar represents rest average. This is very useful for monitoring the infection control practices in the regional centre. Similar graph is available for comparative account of RIS in each department of the regional centre. This graph can be generated for user selected sample type, department and organism species.


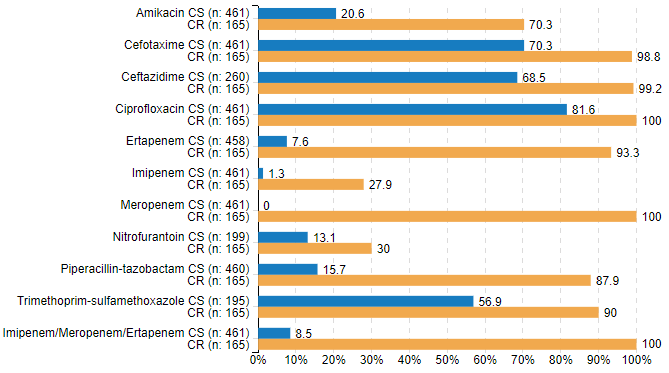


**Figure S13**: Bar charts depicting resistance pattern by Carbapenem resistance and susceptible strains against other antibiotics. For each antibiotic, upper bar represents resistance percentage in carbapenem-susceptible strains and lower bar represents resistance in carbapenem-resistance strains. This can be generated for selected sample type and organism species. A similar graph depicting resistance pattern by methicillin resistance and susceptible strains against other antibiotics is also available.


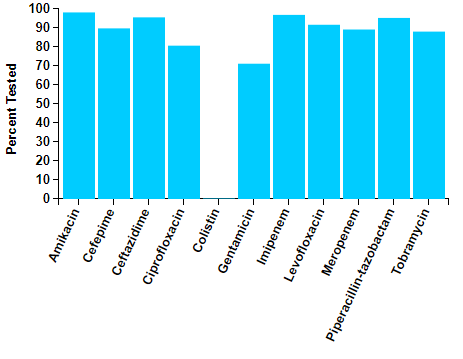


**Figure S14**: Bar charts depicting percentage of antibiotics tested for a user-selected antibiotic panel. This will help in keeping a track of AMST practices across the network as well as within a regional centre.


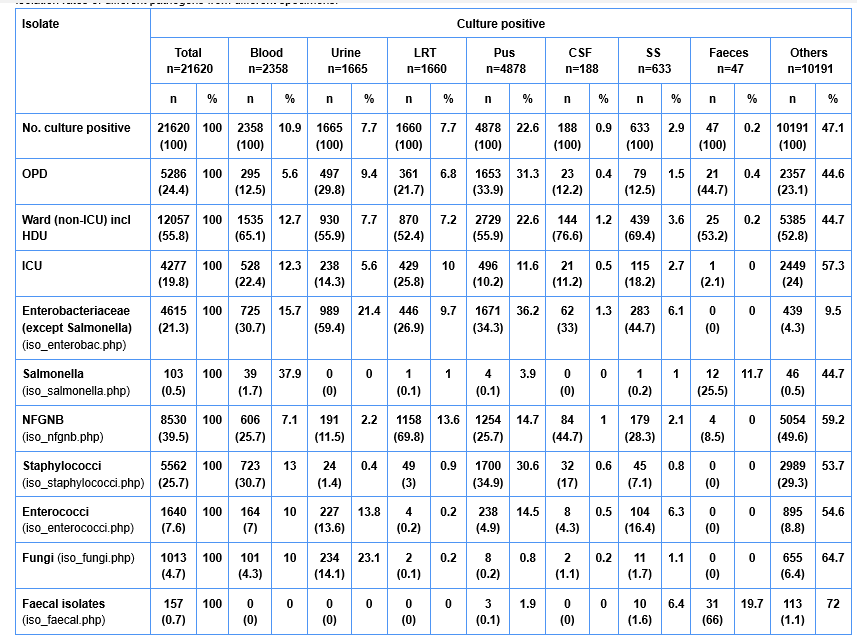


**Figure S15**: Figure depicting screenshots of table generated for isolation percentages of different pathogen groups from commonly tested samples. It also shows the distribution of samples across different locations. Graphics corresponding to this table are available in the figures below.


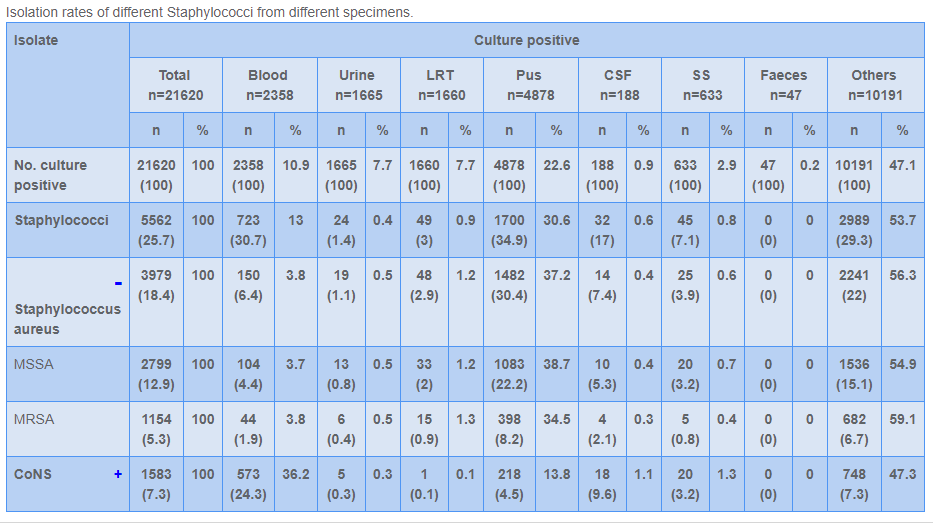


**Figure S16**: Figure depicting screenshots of table generated for isolation percentages of Staphylococcus species from different sample types. Isolation percentages for species of S. aureus and CoNS can be seen by clicking on the ‘+’ sign. Similar tables can be generated for all organism groups in the network.


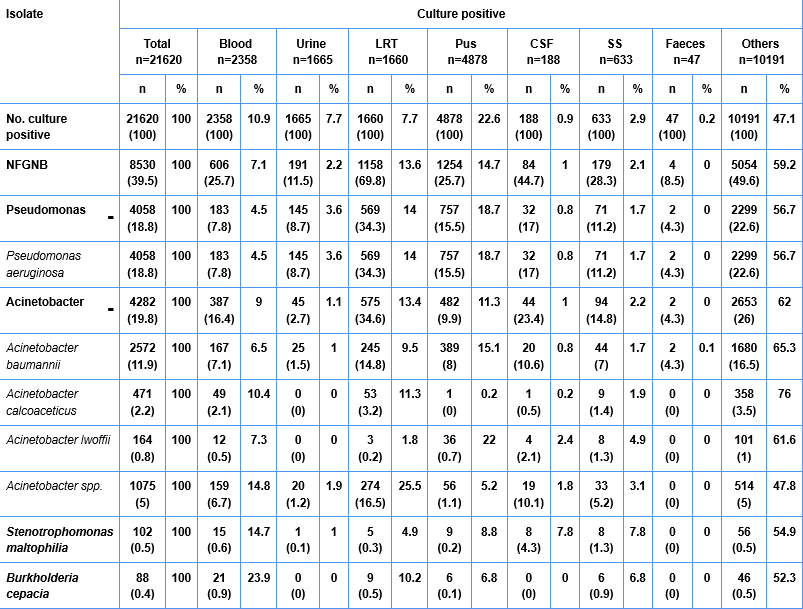


**Figure S17**: Figure depicting screenshots of table generated for resistance percentage of Non-fermenter gram negative bacterial species from different sample types. Similar tables can be generated for all organism groups in the network.


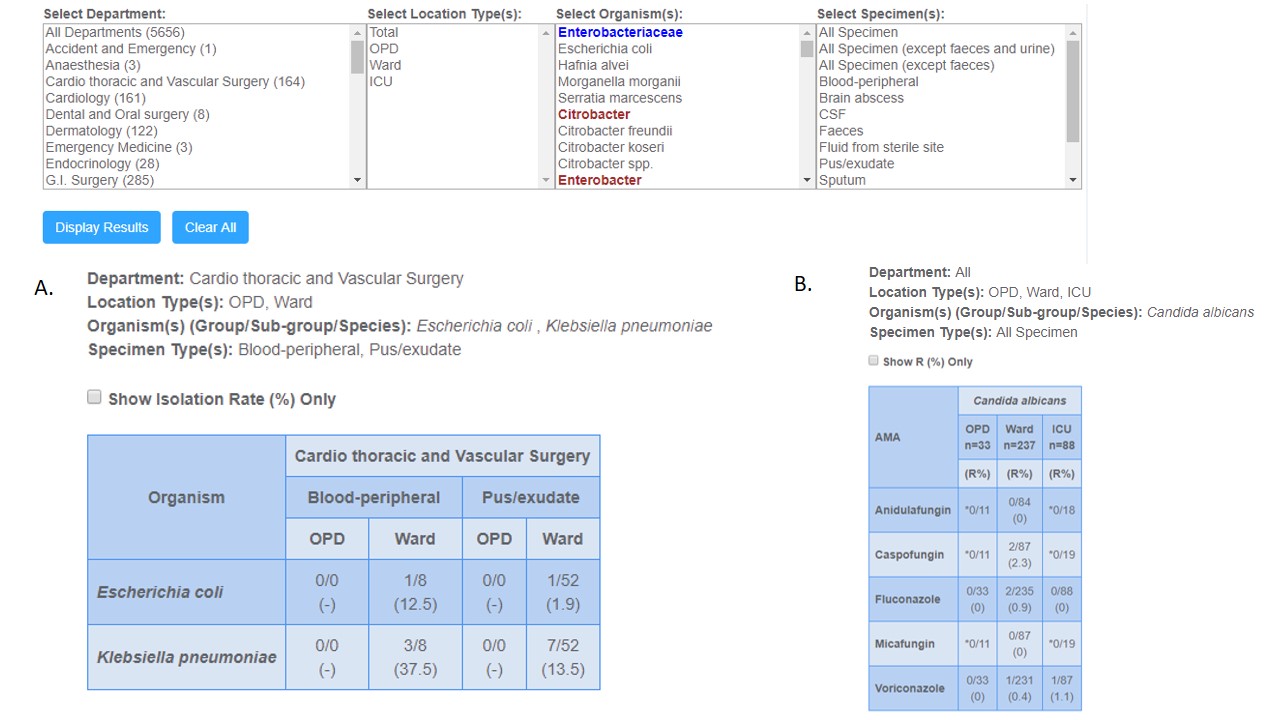


**Figure S18**: Figure representing the options available for generating tables using the advanced analysis option. User can select any combination of department, location, organism and sample to generate tables for isolation rates (A) and resistance pattern (B).


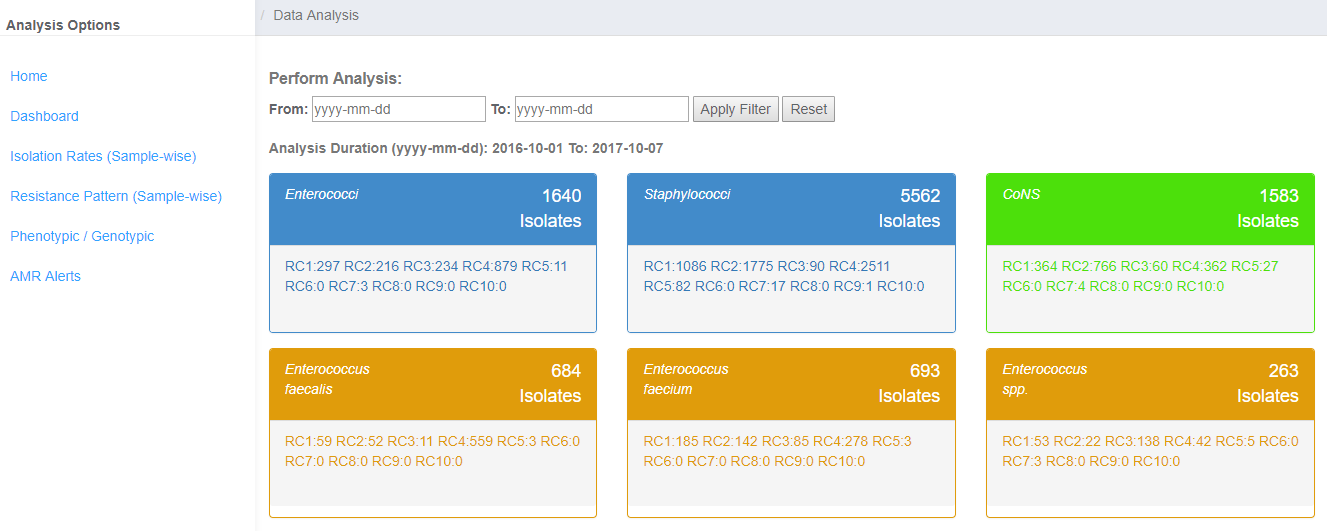


**Figure S19**: Figure representing dashboard for nodal centre analysis module. Broader categories of the available analysis options are shown in the left panel. Dashboard provides number of accepted records for the organism species and groups specific to the nodal centre. Options to select laboratories and duration for analysis is also available on the dashboard.


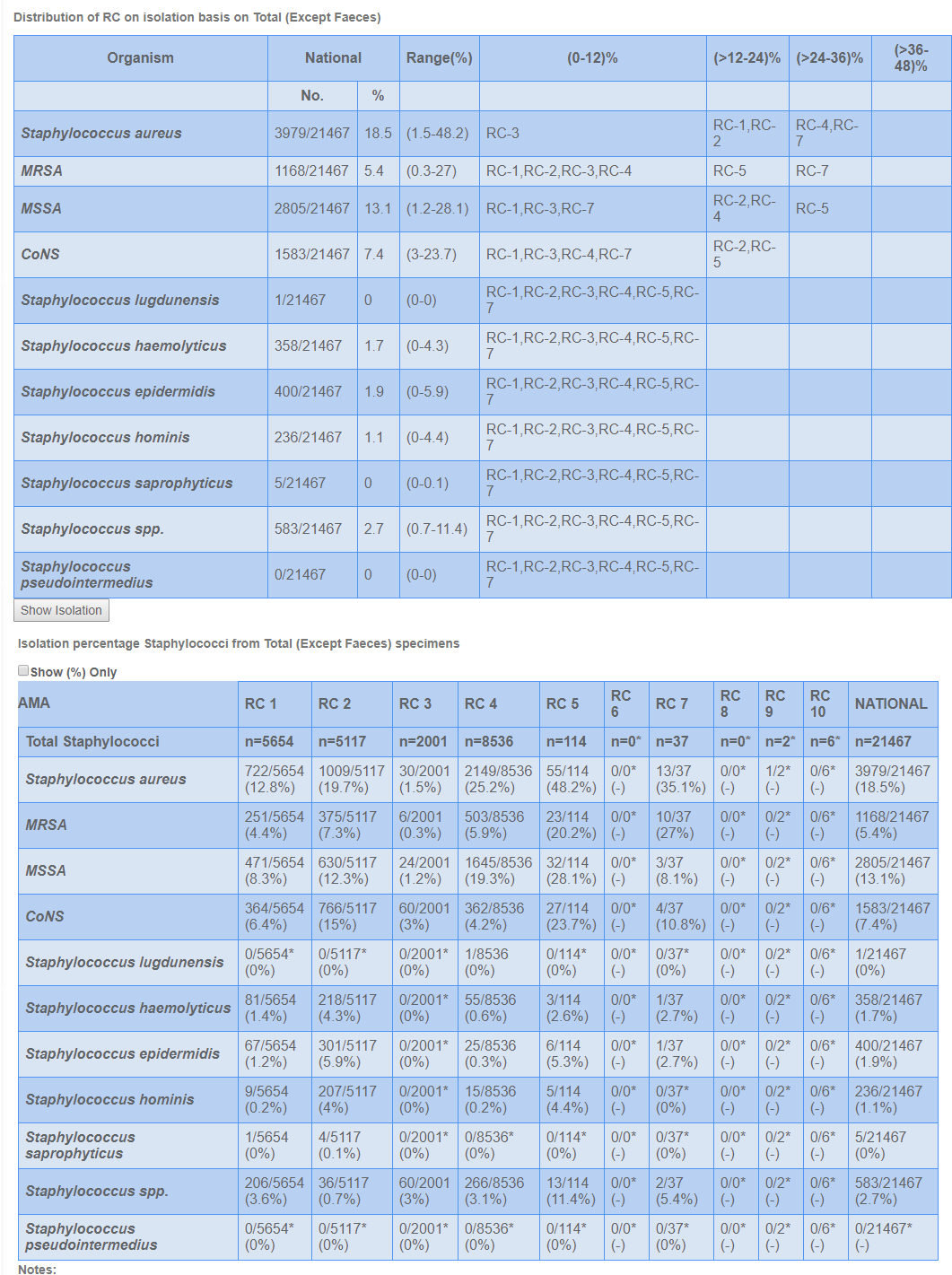


**Figure S20**: Figure showing screenshot of isolation tables for a nodal centre analysis. First table depicts regional centre wise isolation distribution. It will be useful in identifying outliers. The second table gives exact isolation percentages in each regional centre.These are generated for each organism group under the nodal administrator.


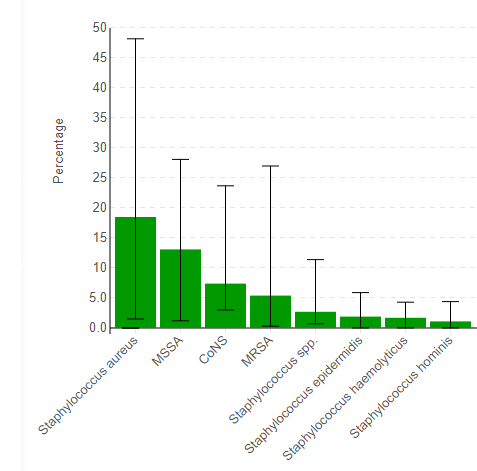


**Figure S21**: Bar graphs representing isolation percentage of Staphylococcus species across all regional centres. This can be generated for all organism groups for a user selected sample type. Bars represent national average and range is from the lowest isolation percentage to the highest in the network.


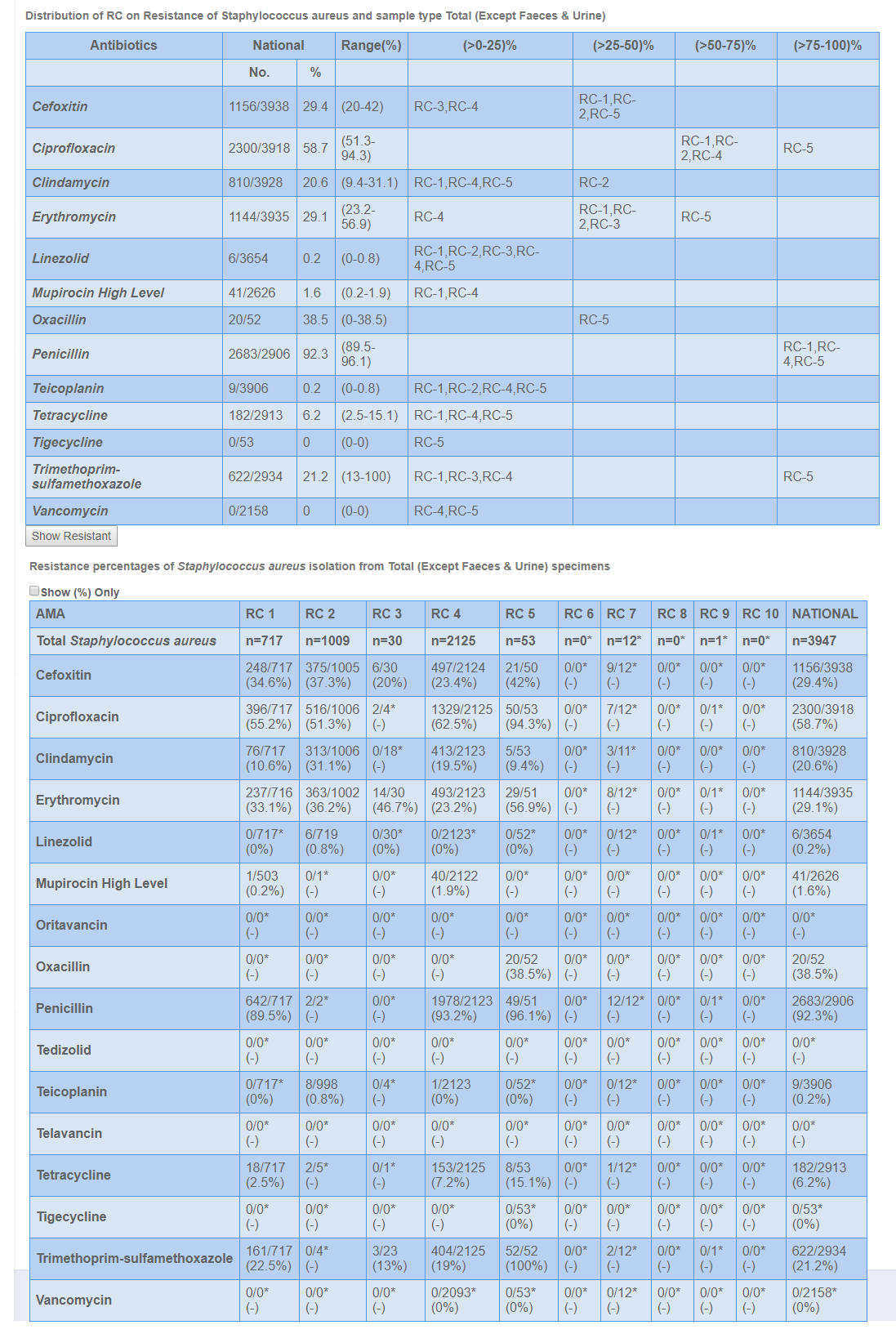


**Figure S22**: Figure showing screenshot of resistance tables for a nodal centre analysis. First table depicts regional centre wise resistance patterns. It will be useful in identifying outliers. The second table gives exact resistance percentages in each regional centre. These are generated for each organism species under the nodal administrator


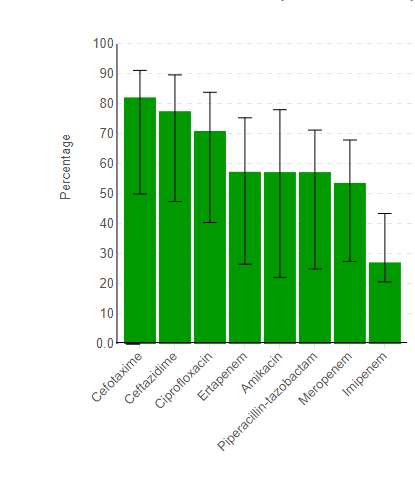


**Figure S23**: Bar graphs representing resistant percentages for Klebsellia pnemonie isolated across all regional centres. This can be generated for all organism groups for a user selected sample type. Bars represent national average and range is from the lowest resisance percentage to the highest in the network.


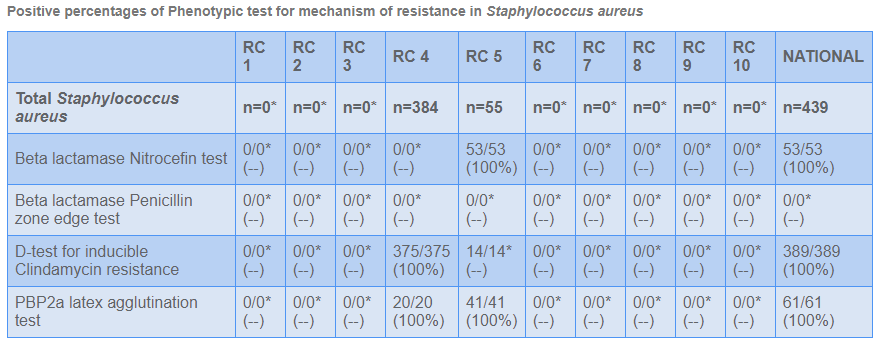


**Figure S24**: Figure showing screenshot of table generated for positive percentages of Phenotypic tests for mechanism of resistance in Staphylococcus aureus. Similar tables are generated for all phenotypic and genotypic tests for mechanism of resistance in all organism species.


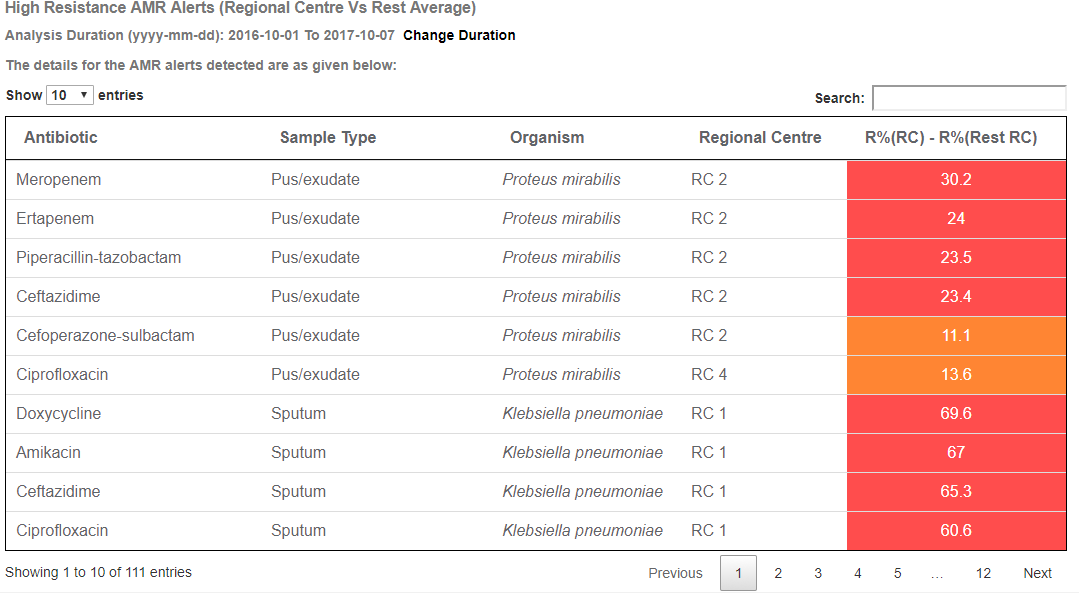


**Figure S25**: Figure depicting higher resistance alerts for the organism species validated by the Nodal administrator showing higher resistance in all regional centres as compared to others. Tool checks in a combination of all samples, antibiotics and organism for higher resistance in the selected Regional centre as compared to others. 10-20% higher resistance is color coded orange and 20% and above resistance is color coded red. Similar alerts are available for each nodal administrator in the network. Also, alerts for lower resistance information is also provided for each nodal centre.
